# Anomaly Detection for Agricultural Vehicles Using Autoencoders

**DOI:** 10.3390/s22103608

**Published:** 2022-05-10

**Authors:** Esma Mujkic, Mark P. Philipsen, Thomas B. Moeslund, Martin P. Christiansen, Ole Ravn

**Affiliations:** 1 Automation and Control Group, Department of Electrical Engineering, Technical University of Denmark, 2800 Kongens Lyngby, Denmark; or@elektro.dtu.dk; 2 AGCO A/S, 8930 Randers, Denmark; martinpeter.christiansen@agcocorp.com; 3 Visual Analysis and Perception Lab, Department of Architecture, Design, and Media Technology, Aalborg University, 9000 Aalborg, Denmark; mpph@create.aau.dk (M.P.P.); tbm@create.aau.dk (T.B.M.)

**Keywords:** anomaly detection, agricultural vehicle, autoencoder, deep learning, computer vision

## Abstract

The safe in-field operation of autonomous agricultural vehicles requires detecting all objects that pose a risk of collision. Current vision-based algorithms for object detection and classification are unable to detect unknown classes of objects. In this paper, the problem is posed as anomaly detection instead, where convolutional autoencoders are applied to identify any objects deviating from the normal pattern. Training an autoencoder network to reconstruct normal patterns in agricultural fields makes it possible to detect unknown objects by high reconstruction error. Basic autoencoder (AE), vector-quantized variational autoencoder (VQ-VAE), denoising autoencoder (DAE) and semisupervised autoencoder (SSAE) with a max-margin-inspired loss function are investigated and compared with a baseline object detector based on YOLOv5. Results indicate that SSAE with an area under the curve for precision/recall (PR AUC) of 0.9353 outperforms other autoencoder models and is comparable to an object detector with a PR AUC of 0.9794. Qualitative results show that SSAE is capable of detecting unknown objects, whereas the object detector is unable to do so and fails to identify known classes of objects in specific cases.

## 1. Introduction

The development of autonomous vehicles that can operate safely in highly unstructured environments, such as agricultural fields, proved to be a complex task. It requires an interdisciplinary approach [1] and needs to address the challenges of natural variation and uncertainty [2]. In order to meet the safety requirements, an autonomous agricultural vehicle needs to be equipped with robust obstacle detection algorithms that run in real time. In general, such an obstacle detection system relies on inputs from multiple sensor modalities in order to provide sufficient information about the surrounding environment and introduce necessary redundancy [3]. A vehicle’s ability to perceive and understand its environment relies heavily on data from cameras. The success of deep learning architectures for image classification, semantic segmentation, and object detection, greatly benefited the application of deep learning in scene perception for autonomous vehicles [4].

Agricultural fields are dynamic, unstructured and diverse environments. Supervised approaches to object detection and semantic segmentation are trained to detect objects from a predefined set of classes. Since these algorithms need substantial data for each class, they are usually limited to detection and classification of only the most common classes of objects that can be encountered in a field. While these approaches are essential for solving higher-level tasks such as scene understanding and autonomous navigation, they do not provide a complete solution for safe operation due to their inability to detect unknown objects. On the other hand, self-supervised approaches, such as autoencoders applied in anomaly detection, are trained to look for patterns that do not conform to normal operating conditions. Therefore, they are able to detect a wide range of objects in the field that pose a potential danger and need to be treated as obstacles. For these reasons, anomaly detection is crucial for developing safe and reliable perception systems for autonomous and semiautonomous agricultural vehicles.

In the agricultural context, all objects in the field that are a potential obstruction to the safe operation of a vehicle would be treated as anomalies. The anomalies include humans, animals, other vehicles, holes, standing water, buildings, and various other objects left in the field intentionally or unintentionally, e.g., different tools and equipment. Developing a system that can warn the machine operator when anomalies need their attention allows the operator to focus more on the other aspects of agricultural operation.

Autoencoders are commonly used in solving anomaly detection tasks and have applications in data compression and feature learning. An autoencoder is composed of two parts: encoder and decoder. The encoder network maps the input data to low-dimensional feature space, while the decoder attempts to reconstruct the data from the projected low-dimensional space [5]. The encoder and decoder are trained together with reconstruction loss functions to minimize the reconstruction error between the input and reconstructions. Using normal data to train the autoencoder enables the model to learn to reconstruct normal data instances from low-dimensional feature spaces with low reconstruction error. Since anomalies deviate from normal data instances, they are much harder to reconstruct from the same low-dimensional feature space, resulting in greater reconstruction error. Therefore, reconstruction error can be used to identify anomalies and generate anomaly maps.

**Figure 1:**
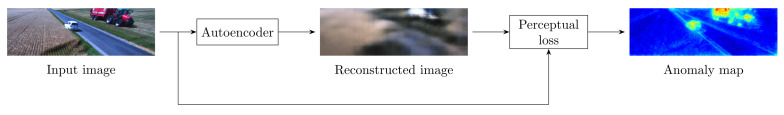
Anomaly detection concept.

This paper investigates the application of different autoencoder variants for the detection of anomalies in an agricultural field. A semisupervised autoencoder (SSAE) trained with max-margin-inspired loss function applied to input image and reconstructed image at pixel level is proposed. The proposed approach is evaluated and shown to outperform the basic autoencoder (AE), the vector-quantized variational autoencoder (VQ-VAE) and the denoising autoencoder (DAE). The anomaly maps are generated using relative-perceptual-L1 loss [6]. The overview of the autoencoder-based anomaly detection concept for all four models is shown in Figure 1. The models are trained on a dataset collected by the front camera of an agricultural vehicle during summer harvest in Denmark. Finally, the performance of autoencoder architectures is compared with the performance of the baseline object detector model based on YOLOv5s [7]. The object detector is trained to detect the classes of objects commonly found in a field during harvesting season.

The paper’s main contributions are as follows:

- Differentt autoencoder models and a baseline object detection model are compared in a difficult task of in-field anomaly detection for autonomous agricultural vehicles using image data. To the best of our knowledge, this is the first analysis of autoencoders applied in the agricultural domain to detect anomalies that pose a potential operational risk for agricultural vehicles.
- The paper introduces a semisupervised anomaly detection strategy that leverages a small number of image samples with labeled anomalies and applies max-margin loss function to reinforce better discrimination of normal and abnormal pixels. The proposed approach outperforms the other investigated autoencoder models.
- The results of the proposed autoencoder model and YOLOv5 model are compared. It is shown that the object detector is challenged in detecting unknown classes of objects as well as the trained classes of objects in some cases.

The remainder of the paper is structured as follows. Section 2 reviews related work for anomaly detection using convolutional autoencoders and deep learning in the agricultural domain for anomaly detection. Section 3 describes the dataset, autoencoder architectures as well as the object detector model. In Section 4, the performance of the trained networks is evaluated. This is followed by the conclusion in Section 5.

## 2. Related Work

Anomaly detection has been applied in several domains, such as fraud detection, medical imaging, Internet of Things (IoT), surveillance and monitoring and time series data analysis [8]. In the agricultural domain, it has been mostly applied in precision farming [9,10,11,12,13] and to a far lesser extent in navigation and obstacle detection [14,15].

Earlier work by Christiansen et al. [14] exploited the homogeneous characteristics of an agricultural field and combined CNN and background subtraction algorithms. The work demonstrated successful use of background subtraction for a moving camera in agriculture, exploiting the fact that images taken from a front camera of an agricultural vehicle moving along rows in the field are similar. This approach is able to detect heavily occluded, distant and unknown objects. In addition, the approach showed better or comparable results with state-of-the-art object detectors in the agricultural context.

The image resynthesis methods focus on finding differences between the input image and the image resynthesized from the predicted semantic map. In the work presented by Lis et al. [16], an exiting semantic segmentation algorithm generates the semantic map. Then, the approach utilizes generative adversarial network (GAN) to generate the resynthesized image. Finally, an anomaly map is yielded by a trained discrepancy network that takes the original image, resynthesized image and predicated semantic map as inputs. Ohgushi et al. [17] addressed the detection of road obstacles in road scenes with a complex background where there is a risk of unknown objects that are not present in the training dataset. This study proposed a road obstacle detection method using an autoencoder consisting of modules for semantic segmentation and resynthesized image generation. First, the semantic segmentation network is trained with data from normal road scenes. Next, resynthesized images are created using a photographic image synthesis technique. The method then calculates the perceptual loss between the input and resynthesized images and multiplies it by the entropy for the semantic map to generate an anomaly map. Finally, the method localizes road obstacles and assigns obstacle scores at the superpixel level in the postprocessing step.

A new anomaly detection approach for high-resolution medical data, based on autoencoders with perceptual loss and progressive growing training, was introduced by Shvetsova et al. [18]. Since anomalies in the medical images often resemble normal data, low-quality reconstructions from autoencoders may not capture the fine detail necessary for anomaly detection. This limitation is addressed by training the model with progressive growing technique where layers are added to the autoencoder and the depth of the features in perceptual loss is increased during training.

Van Den Oord et al. [19] introduced a new generative model VQ-VAE. The model combines VAEs with vector quantization to obtain a discrete latent representation. The output of the encoder is mapped to the nearest embedding vector from the shared discrete embedding space. The corresponding embedding vector is used as the input to the decoder. The model parameters consist of encoder network, embedding space and decoder network. In order to avoid the unwanted reconstructions of anomalies, Wang et al. [20] used a discrete probability model to estimate the latent space of the autoencoder and exclude the anomalous components of the latent space. More specifically, the VQ-VAE model is trained on normal data to obtain the discrete latent space of normal samples. Then, the deep autoregressive model PixelSNAIL [21] is used to estimate the probability distribution of the latent space. The deviating components of the latent space are resampled from the distribution and decoded to yield a restored image.

Vincent et al. [22] proposed a denoising autoencoder. The proposed training principle makes the learned representation robust to partial corruption. The input is first corrupted with noise, and the model is trained to reconstruct the original input from the corrupted one. Tun et al. [23] used a convolutional autoencoder for denoising of outdoor facial images. The convolutional denoising autoencoder was used for efficient denoising of medical images by Gondara [24]. The results showed that a small training dataset is sufficient for good denoising performance.

In addition to a large unlabeled dataset of normal data, weakly supervised anomaly detection uses a small labeled dataset during training to improve detection. Ruff et al. [25] introduced an end-to-end deep methodology for semisupervised anomaly detection called deep semisupervised anomaly detection (DeepSAD). Zhou et al. [26] leveraged unsupervised anomaly detection based on an autoencoder to extract feature representations of normal data. The extracted feature representation is used for weakly supervised anomaly detection.

An essential step in anomaly localization in image data is the visualization of detected anomalies. Baur et al. [27] generated an anomaly map by computing the pixelwise L1-distance between an input image and image reconstruction by autoencoder. Then, the resulting residual is thresholded to obtain a binary segmentation. On the other hand, Lis et al. [16] relied on a discrepancy network trained to detect significant image discrepancies. The discrepancy network uses the original image, the predicted semantic labels and the resynthesized image as inputs. A pretrained VGG [28] network is used to extract features from the input image and resynthesized image, while a custom CNN network is used to extract features from predicted semantic labels. The features of all the streams are concatenated and fused using 1 × 1 convolutions at each level of the feature pyramid. The final discrepancy map is generated by passing features and their correlations through the decoder network.

Although different anomaly detection approaches have been successfully applied in various domains, very little research has been conducted on anomaly detection for autonomous agricultural vehicles. Moreover, the current research does not indicate which autoencoder architecture performs the best for this use case scenario. Hence, the focus of this paper is to investigate and compare several autoencoder models.

## 3. Materials and Methods

This section presents the datasets used for training and testing the models, followed by the description of autoencoder models and the baseline model.

### 3.1. Dataset Description

In order to train and evaluate all the models, several datasets were required, as listed in Table 1. Currently, no relevant datasets are publicly available for the agricultural domain. Images in the datasets for training and evaluation of autoencoders are resized to 800 × 160. In the datasets for training and evaluation of the baseline model, images are resized to 640 × 128. Each dataset is described in more detail below.

#### 3.1.1. Normal Dataset

AE and VQ-VAE use a dataset with images depicting normal operating conditions. The dataset consists of 2344 images collected over 9 days of summer harvest in Denmark.

#### 3.1.2. Synthetic Dataset

DAE requires image pairs of an image with anomalies and its normal equivalent. Collecting such pairs of images in the real world is not possible for this application. Therefore, one way of creating such a paired dataset is to have a dataset with annotated objects and use the object mask to add those objects to normal images. In this way, the training of DAE, in addition to the dataset with normal images, requires an annotated dataset of anomaly objects at the pixel level. The previously described normal dataset is used as the set of background scenes. A separate annotated dataset, collected along with the normal dataset, is used to extract the objects that present obstacles and, in this context, anomalies. The placement of the object in the new background scene is kept the same as in the original image. Finally, the Gaussian blur with 5 × 5 kernel is used to blend the object into the scene and mitigate boundary artifacts.

#### 3.1.3. Object Detection Dataset

For the training of the baseline model, a dataset annotated for box-based object detection is used. The dataset consists of 7.9 k images collected by two agricultural vehicles over 13 days. The annotated classes are: tractor, combine, combine header, grain wagon, square bale, person and car.

#### 3.1.4. Test Dataset

The annotated test dataset consists of 1994 images in total. Out of those images, 645 images represent normal data, and 1349 images are images with objects collected on the same days as the normal dataset. In addition, the images are annotated with non-overlapping instance segmentation and bounding boxes.

### 3.2. Autoencoders

This section provides an overview of autoencoder architectures and the anomaly map generation method.

#### 3.2.1. AE

**Figure 2:**
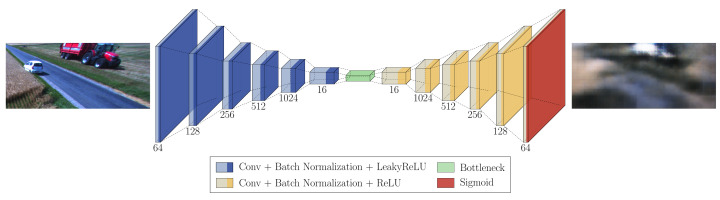
Illustration of the basic autoencoder architecture applied to image reconstruction task in agricultural environment. The input of the network is an RGB image of a field scene. The output is the corresponding reconstructed image.

In order to reconstruct normal data and use reconstruction error as an anomaly score, a basic autoencoder model was trained. The architecture of the network is illustrated in Figure 2. The model consists of an encoder network that compresses input data and latent space and a decoder network that reconstructs data from the compressed representations. The encoder network has 6 convolutional layers with an increasing number of filters (64, 128, 256, 512 and 1024). The decoder network corresponds to the encoder with 6 convolutional layers with a decreasing number of filters (1024, 512, 256, 128 and 64). The encoder network and decoder network share a bottleneck with 16 channels. Each convolutional layer, with the exception of the final layer, is followed by batch normalization and LeakyReLU as activation function. The final convolutional layer is followed by sigmoid activation. All convolutional layers in the encoder use stride 2 to downsample the feature maps, except for the final convolution layer that uses stride 1. Likewise, the decoder network uses transposed convolutions with stride 2, except the first layer with stride 1, to upsample input feature maps. Using strided convolution layers enables downsampling and upsampling functions to be learned simultaneously with the rest of the network. The autoencoder is trained using images from a normal dataset. Mean squared error (MSE) between the input image and predicted image is used as a loss function. The anomaly map is generated by calculating relative-perceptual-L1 loss between the input image and reconstructed image.

#### 3.2.2. VQ-VAE

The VQ-VAE architecture, as presented in [19], is trained using the normal dataset. This represents the first step of the training process. Afterwards, all images from normal training datasets are encoded using the trained VQ-VAE to collect a latent code set, and the probability distribution of this latent code set is estimated using Gated-PixelCNN. At the prediction stage, the model yields two output images. The first image is the reconstructed image decoded from the original latent set. Then, when the latent code of an input image is out of the distribution learned in the second step, Gated-PixelCNN conducts resampling operations on it. Finally, the resampled latent code is decoded as a restored image, which is used for anomaly detection by calculating the relative-perceptual-L1 loss with the directly reconstructed image.

#### 3.2.3. DAE

A denoising autoencoder has the same architecture as AE, a 6-layer encoder and a 6-layer decoder that share a 16-channel bottleneck. The model is trained using a synthetic dataset. The input to the network is images containing anomalies, and the targets are corresponding images without anomalies. The loss function used for training is MSE between the target and predicted images. In this way, the autoencoder network learns to remove anomalies from the input image. The anomaly map is generated by calculating relative-perceptual-L1 loss between the input and predicted images with anomalies removed.

#### 3.2.4. SSAE

A semisupervised autoencoder has the same structure as the AE and DAE models. The model is trained using semisupervised learning with a training dataset consisting of the normal dataset and 500 abnormal samples with labeled anomalies.The input to the network are images from the training dataset, with and without anomalies, and the output of the network is corresponding reconstructed images. In addition to minimizing the reconstruction error, the objective function should distinguish between normal and abnormal samples. This is accomplished by using ground-truth masks for images to identify the pixels that should be classified as normal or abnormal. Then, the corresponding loss term between input image *x* and predicted reconstructed image *y* is calculated according to:

(1) $$L\left(x,y\right)=\frac{1}{N}\sum_{i}^{N}∥x_{i}-y_{i}{∥}_{2}+\max(0,a_{0}-\frac{1}{M}\sum_{i}^{M}∥x_{i}-y_{i}{∥}_{2}),$$

where *N* is the total number of normal pixels in the image, *M* is the total number of abnormal pixels, $x_{i}$ is *i*th pixel value in the input image, and $y_{i}$ is the value of the corresponding pixel in the reconstructed image.

By introducing max-margin term in the loss function, reconstruction error for abnormal pixels affect the total loss only if it is lower than $a_{0}$. As a result, the reconstruction error is kept above the threshold for abnormal samples. Threshold $a_{0}$ is determined experimentally in Section 4.1. The anomaly map is generated by calculating relative-perceptual-L1 loss between the input image and reconstructed image.

#### 3.2.5. Anomaly Map Generation

The anomaly scores for each pixel and anomaly map are calculated by relative-perceptual-L1 loss. The VGG19 [28] network, pretrained on ImageNet dataset for the object classification task, is applied to the input image and the reconstructed image. Then, the obtained deep features of hidden layers are normalized by the mean and standard deviation of filter responses precalculated on ImageNet. The L1-norm is calculated between the normalized features and divided by the average values of these features. For example, let f(x) be the feature map obtained from a hidden layer of the network applied to image *x*. Then,

(2) $$\tilde{f}\left(x\right)=\frac{f(x)-\mu }{\sigma }$$

is the normalized feature map where μ and σ are the precalculated mean and standard deviation of filter responses. The relative-perceptual-L1 loss between image *x* and *y* is defined as

(3) $$L\left(x,y\right)=\frac{∥\tilde{f}\left(x\right)-\tilde{f}{\left(y\right)∥}_{1}}{∥\tilde{f}{\left(x\right)∥}_{1}}.$$

In this paper, the loss is obtained from outputs of the second convolutional layers of 5 convolutional blocks in VGG19.

#### 3.2.6. Anomaly Score per Image

After obtaining the anomaly map, anomaly scores for each pixel need to be classified as normal or abnormal in order to identify the areas with anomalies. In other words, a suitable threshold value needs to be found. In order to do this, anomaly maps of images from the test set are thresholded by a range of threshold values. Then, the thresholded anomaly maps are compared with the ground truth, and the threshold that yields the maximum mean intersection over union (IoU) with the ground truth is selected. The determined threshold values for the implemented autoencoders can be found in Table 2.

Finally, the total anomaly score for the image is calculated as the percentage of pixels with an anomaly score above the threshold value.

This anomaly score is used to optimize the F1 score on the test set and determine the corresponding threshold for classifying images as normal or abnormal according to the Equation (4).

(4) $$\left\{\begin{array}{cc} \mathrm{anomaly}\_\mathrm{score}<\mathrm{threshold}, & \mathrm{normal}\\ \mathrm{anomaly}\_\mathrm{score}\geq \mathrm{threshold}, & \mathrm{abnormal} \end{array}\right.$$

The threshold values are listed in Table 2.

### 3.3. Baseline Model

The lightest model version of YOLOv5, YOLOv5s, was chosen as the baseline model. The model is a single-stage object detector and consists of a backbone network, neck and detection head. The cross stage partial connections (CSP) backbone network is used to extract features from the input image. The layers in the neck of the network are inserted to collect feature maps from different stages. This enables the model to perform better when detecting objects of various sizes and scales. In YOLOv5, PANet is used as the neck network to obtain feature pyramids. The YOLO head proposed in YOLOv3 [29] is used to perform the final detection part. It applies anchor boxes on features and generates final output vectors with class probabilities, objectness scores and bounding boxes. In YOLOv5s, the SiLU activation function is used in hidden layers, while the final detection layer uses the sigmoid activation function.

## 4. Results and Discussion

Results are divided into two subsections. The first section shows experimental results for determining the optimal threshold $a_{0}$ for the objective function of SSAE. The second section presents a performance evaluation of trained models followed by qualitative examples comparing the performance of the autoencoders and the object detector. The training hyperparameters for all models are listed in Table 3.

### 4.1. Experimental Results for $a_{0}$ Threshold

**Figure 3:**
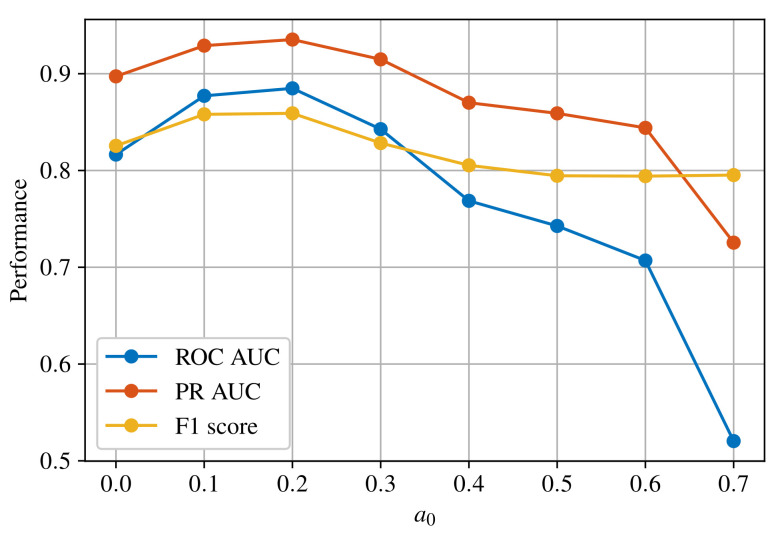
Performance comparison of SSAE models for different values of $a_{0}$.

Threshold $a_{0}$ represents the margin that separates normal and abnormal pixels in the training set. Setting this threshold to a low value prevents the model from discriminating between normal and abnormal samples. On the other hand, setting this threshold to a large value makes the optimization task very difficult, and the model’s performance might start to deteriorate. Therefore, the model is trained for different values of $a_{0}$ and the optimal one is determined based on the results. Figure 3 shows the performance comparison for three different metrics.

With a basis in Figure 3, the optimal threshold is found to be $a_{0}=0.2$ for the proposed semisupervised autoencoder model.

### 4.2. Performance Evaluation of Models

Due to the inconsistent outputs of the models, each model is evaluated against the annotated dataset according to its ability to classify an image as anomalous or not. For autoencoders, anomaly scores are computed for each image, and images are classified according to Equation (4) for various thresholds. For the baseline model, if objects are detected in an image, the image is classified as anomalous.

**Figure 4:**
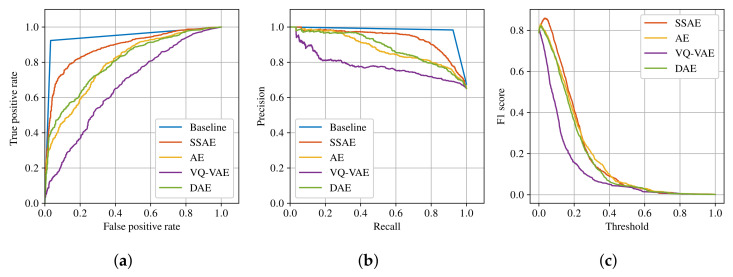
Performance comparison of the autoencoder models SSAE, AE, VQ-VAE and DAE and the baseline YOLOv5. (**a**) ROC curve. (**b**) Precision/recall curve. (**c**) F1 score.

A receiver operating characteristic (ROC) curve, precision/recall curve and F1 score are generated for each model in Figure 4. Furthermore, the models’ performances are measured using the maximum F1 score, the area under the curve for ROC curve (ROC AUC) and the area under the curve for precision/recall curve (PR AUC). ROC is one of the most commonly used evaluation metrics for classification tasks and it is insensitive to class imbalance [30]. Since the test dataset is imbalanced, PR metrics are also computed, which provide a more informative measure in the case of imbalanced data [31].

The results can be seen in Table 4. The baseline model has the highest performance scores on all three metrics. The proposed SSAE has the highest performance scores from the autoencoder models, followed by AE and DAE with similar performances and VQ-VAE with the worst performance of the autoencoder models.

The generated ROC curves and precision/recall curves are shown in Figure 4a,b, respectively. For the autoencoder models, the generated F1 score curves with normalized thresholds are also shown in Figure 4c. The ROC curve and precision/recall curve show that SSAE has the best performance compared with the other autoencoder models.

**Figure 5:**
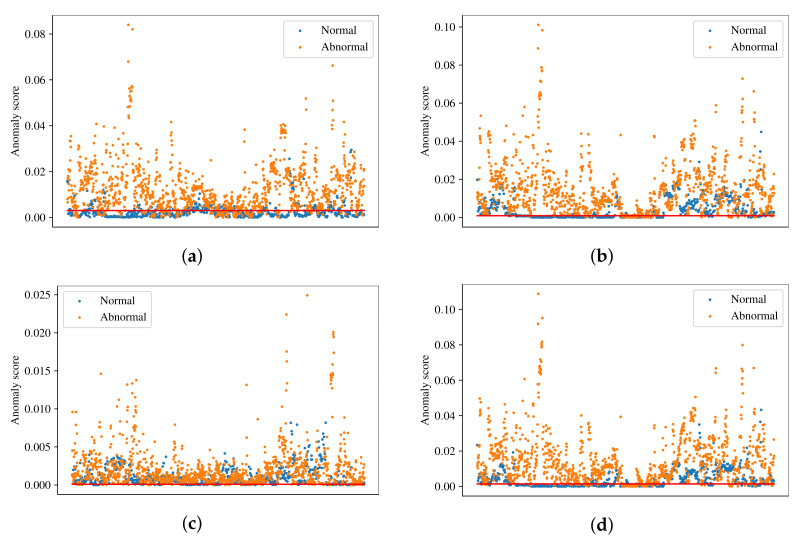
Distribution of normal and abnormal samples from the test set. (**a**) SSAE, thr=0.003. (**b**) AE, thr=0.0009. (**c**) VQ-VAE, thr=0.0001. (**d**) DAE, thr=0.0014.

The distribution of normal and abnormal samples in the test dataset is shown in Figure 5. In the figure, blue dots correspond to normal samples, orange dots correspond to abnormal samples and red lines represent the thresholds found by optimizing the F1 score. For a good anomaly detector, normal samples should be placed below the threshold line and abnormal samples above the threshold line. The results in Figure 5 support the performance results stated earlier. It can be clearly seen in Figure 5c that VQ-VAE has trouble classifying normal and abnormal samples, since the anomaly scores of a significant number of those samples fall within the same range. However, abnormal samples have higher anomalies scores in general. On the other hand, Figure 5b,d shows that AE and DAE have a good ability of distinguishing between normal and abnormal samples. The SSAE has the best ability to distinguish between the samples, where the majority of normal samples is below threshold and the majority of abnormal samples is above threshold.

**Figure 6:**
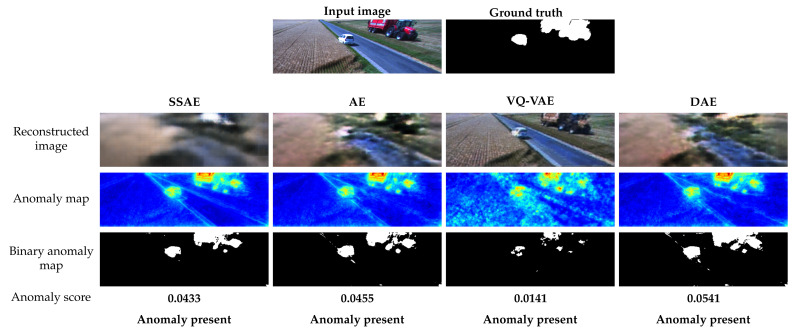
Anomaly detection example for autoencoders. The example input image shows a tractor with a trailer and a car in the field. The anomaly maps are generated by relative-perceptual-L1 loss and thresholded for each autoencoder to obtain a binary anomaly map. The calculated anomaly scores are above thresholds and the image is correctly classified as anomalous.

The qualitative performance of the autoencoder models is presented in Figure 6. The example image is captured by the front camera of the combine harvester during field operation. In this scenario, the tractor with a trailer and the car are potential obstacles in the harvester’s path and should be detected as anomalies. The reconstruction images show that the different autoencoders are reconstructing the images as expected. For SSAE, there is significant difference in the reconstruction of normal and abnormal areas. The AE and VQ-VAE models are reconstructing anomalies poorly, while DAE is able to remove the anomalies from the image. The anomaly maps of SSAE, AE and DAE show the anomalies clearly. However, the anomaly map produced by SSAE has smoother normal areas. The anomaly map generated by VQ-VAE has significantly more noise in areas without anomalies. The anomaly scores are above the threshold for all four autoencoders, indicating that the image would be correctly classified as anomalous in all cases.

**Figure 7:**
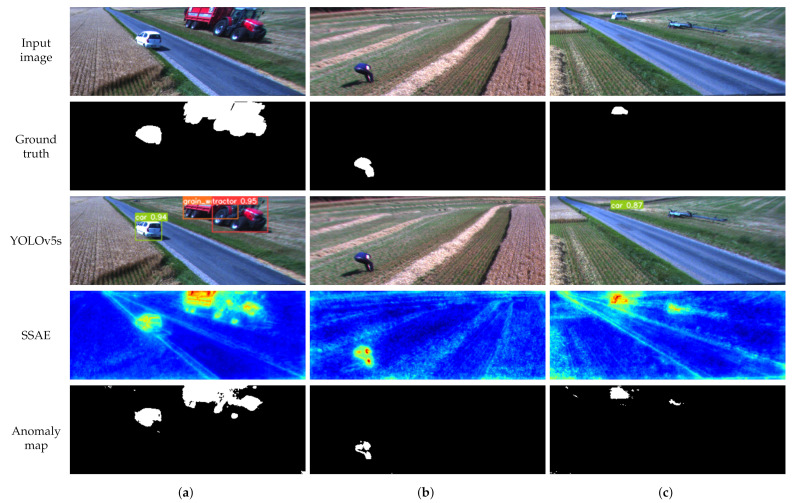
Detection examples. (**a**) Object detector and autoencoder detect objects in the image; (**b**) object detector fails to detect a human; (**c**) autoencoder detects a car and an object that is not part of the annotated dataset, while the object detector detects only the car.

Comparing anomaly detectors with the baseline YOLOv5 model, it can be seen from Figure 4 and Table 4 that SSAE has the closest performance to the baseline model. One major difference between the anomaly detectors and object detector is that anomaly detectors do not provide class labels for the detected objects. Another major difference is the requirements for the training dataset. For example, AE and VQ-VAE require only normal data that does not need to be annotated. On the other hand, SSAE requires a small number of annotated samples. Furthermore, DAE requires annotated masks for objects, which can be time-consuming. Although object detectors provide class labels for anomalous objects, they require a large amount of data for each object class. Given that the agricultural fields are highly unstructured environments, it is unrealistic to collect enough data for each object that could potentially be found in a field. For example, Figure 7c shows a header trailer that was not included as a class for annotation. YOLOv5s cannot detect it as an object, but SSAE can detect it as an anomaly. Moreover, Figure 7b shows a scenario where YOLOv5s fails to detect a person because of their body position. In the same scenario, SSAE is able to detect a person as an anomaly successfully.

### 4.3. Potential Applications

The proposed anomaly detection can be integrated with agricultural vehicles at several levels of driving automation.

As a first step towards autonomy, the proposed anomaly detection can be integrated with current agricultural vehicles as an assisted tool. The current generation of agricultural vehicles use commercially available products, such as autosteering and tractor-guidance systems, to navigate automatically and more efficiently. However, the human operator is still responsible for obstacle detection and the safety of local navigation. Moreover, the operator monitors the parameters related to the specific agricultural operation and ensures optimal performance. The proposed anomaly detection can be integrated as an assisted tool which provides visual feedback and/or an audio warning to the operator if an anomaly is detected near the vehicle. By providing assistance in detecting objects that may cause a collision, the system relieves the operators of some responsibility and enables them to focus more on monitoring process-related performance.

In the future, the proposed system can be integrated with a fully autonomous agricultural vehicle. According to the functional architecture of the autonomous driving system outlined in [32], the proposed system would be integrated as part of the perception functional block. In general, the perception block receives data from multiple sources and generates a representation of the vehicle’s environment. This information is passed to the planning and decision block for navigation planning as well as reactive behavior. The motion and vehicle control block includes execution of the planned trajectory with movement commands and control of the actuators. The overall robustness of such a complex system and its ability to be certified for autonomous operation depend on the performance of numerous components.

On the environment perception part, and more specifically the processing of image data and object detection, the robustness of the system can be improved by combining multiple approaches to object detection. For example, the proposed anomaly detection would be applied to detect a wide range of potential obstacles without providing any additional information about them, such as their class. On the other hand, an object detector could classify a limited number of those detected obstacles depending on how extensive the training dataset is. Together, the two algorithms would provide the planning and decision system with more complete information about the objects in the vehicle’s environment.

## 5. Conclusions and Future Work

This work presented the application of different autoencoder architectures for anomaly detection in agricultural fields. A Semisupervised autoencoder (SSAE), basic autoencoder (AE), vector-quantized variational autoencoder (VQ-VAE) and denoising autoencoder (DAE) were successfully implemented and evaluated. The performance of the autoencoders was compared with the baseline object detector model trained on an agricultural dataset. SSAE showed performance close to the object detector with a PR AUC of 0.9353 compared with 0.9794 for the object detector. AE and DAE showed lower performance with a PR AUC of 0.8786 and 0.8861, respectively. VQ-VAE had the worst performance with a PR AUC of 0.7797. Even though an object detector can provide valuable information for the object class, examples showed that it could fail in critical cases. In those scenarios, the autoencoder successfully detected objects as anomalies.

The potential applications for the above-mentioned anomaly detection technologies in the agricultural industry are broad. Agricultural vehicles such as combines, forage harvesters, sprayers and tractors with implements such as cultivators, sprayers, seeders and spreaders will, with the current labor shortage, move towards more autonomous operation in the upcoming years. The automated ability to detect abnormal conditions in the forward-moving direction and after field processing is a building block in enabling such future autonomous operating systems.

Future work will investigate the possibility of including temporal information and leveraging anomaly detection from previous image frames for better performance. The work could be further expanded by including domain-specific knowledge such as the type of crop and season and weather conditions. This would ensure a better definition of boundary conditions for a specific model and help identify when it is necessary to collect more data.

## Figures and Tables

**Table 1:** Overview of datasets used for the training of autoencoders and baseline models.

Model	Training Dataset	Evaluation Dataset
SSAE	Normal dataset + 500 abnormal samples	Test dataset (normal data + abnormal data) with pixel-level annotations
AE	Normal dataset	
VQ-VAE	Normal dataset	
DAE	Synthetic dataset (normal data + segmentation masks of objects)	
Baseline YOLOv5	Object detection dataset with bounding box annotation	Test dataset (normal data + abnormal data) with bounding box annotations

**Table 2:** Anomaly thresholds for autoencoder anomaly maps.

Model	Anomaly Map Threshold	mIoU	Anomaly Score Threshold
AE	1.3	0.66	0.0009
VQ-VAE	1.1	0.63	0.0001
DAE	1.2	0.67	0.0014
SSAE	1.4	0.69	0.0030

**Table 3:** Training parameters.

	Autoencoders	YOLOv5s	Comments
Epochs	500	300	Training of all models converges within the specified number of epochs.
Learning rate	$1\times 10^{-5}$	(0.01, 0.1)	
Optimizer	Adam	SGD	
Momentum	(0.9, 0.999)	0.937	
Weight decay	0	0.0005	
Batch size	32	16	
Image size	800 × 160	640 × 128	Images used for training of YOLOv5s are resized to match default input image size and preserve the aspect ratio of images used for training of autoencoders.
Training dataset size	1408 (1708)	3688	Dataset for training of SSAE contains additional abnormal samples.

**Table 4:** Comparison of three autoencoder models AE, DAE and VQ-VAE and baseline YOLOv5s model.

Model	F1 Score	ROC AUC	PR AUC
AE	0.8310	0.7954	0.8786
VQ-VAE	0.7977	0.6661	0.7797
DAE	0.8215	0.8018	0.8861
SSAE	0.8591	0.8849	0.9353
YOLOv5s	0.9526	0.9455	0.9794

## Data Availability

The data presented in this study are available on request from the corresponding author.
